# Diagnostic Performance of ^68^Ga-FAPI PET/CT Versus ^18^F-FDG PET/CT in Evaluating Neoadjuvant Therapy Pathological Response in Breast Cancer

**DOI:** 10.1038/s41523-026-00983-4

**Published:** 2026-06-02

**Authors:** Siji Zhu, Yihua Jin, Chao Hu, Yanli Fan, Xiaochun Fei, Jiajia Hu, Xiaosong Chen

**Affiliations:** 1 Department of General Surgery, Comprehensive Breast Health Center, Ruijin Hospital Affiliated to Shanghai Jiao Tong University School of Medicine, Shanghai, 200025 China; 2 Department of Nuclear Medicine, Ruijin Hospital Affiliated to Shanghai Jiao Tong University School of Medicine, Shanghai, 200025 China; 3 Department of Pathology, Ruijin Hospital Affiliated to Shanghai Jiao Tong University School of Medicine, Shanghai, 200025 China

**Keywords:** Biomarkers, Cancer, Diseases, Medical research, Oncology

## Abstract

Accurate pathological response assessment of breast cancer following neoadjuvant therapy (NAT) is clinically critical but lacks robust imaging methods, so this study evaluated the diagnostic accuracy of ^18^F-fluorodeoxyglucose (FDG) PET/CT and ^68^Ga-fibroblast activation protein inhibitor (FAPI) PET/CT in clinically node positive (cN+) breast cancer patients receiving NAT. This prospective study enrolled pathologically confirmed breast cancer patients who underwent NAT and both PET/CT scans preoperatively. The maximum standardized uptake value (SUVmax) was measured and an optimal ^68^Ga-FAPI PET/CT threshold was determined via receiver operating characteristic (ROC) curve analysis. Among 75 patients, 33 (44.0%) achieved pathological complete response (pCR). SUVmax was significantly lower in the ypT0/Tis group compared to the ypT+ group for both ^18^F-FDG PET/CT (1.45 ± 0.55 vs. 5.19 ± 4.69, *p* < 0.001) and ^68^Ga-FAPI PET/CT (1.59 ± 1.22 vs. 10.45 ± 8.54, *p* < 0.001). In the overall cohort, ^68^Ga-FAPI PET/CT demonstrated higher sensitivity in detecting residual disease compared to ^18^F-FDG PET/CT (0.8 vs. 0.6, *p* = 0.003), with comparable specificity (0.9 vs. 0.9, *p* = 1.00). Exploratory subgroup analysis found that ^68^Ga-FAPI PET/CT had superior sensitivity compared to ^18^F-FDG PET/CT in the hormone receptor-positive (HR + ) group (0.8 vs. 0.6, *p* = 0.02). These findings show that ^68^Ga-FAPI PET/CT outperforms ^18^F-FDG PET/CT in identifying residual breast lesions after NAT in cN+ patients, particularly among HR+ patients, which would guide further treatment strategies after NAT.

## Introduction

Neoadjuvant therapy (NAT) plays a pivotal role in the comprehensive management of breast cancer^1^, not only by downstaging tumors and improving operability, but also by allowing in vivo assessment of treatment efficacy and guiding further escalation of adjuvant therapy^2^. Accurate evaluation of residual disease after NAT is therefore essential for guiding subsequent surgical and systemic treatment decisions^3^.

Conventional imaging modalities such as magnetic resonance imaging (MRI) and breast ultrasound (BUS) are routinely used to evaluate tumor response after NAT^4^. MRI offers high soft-tissue contrast and is currently one of the most reliable methods for estimating residual tumor size^5^. However, it primarily reflects morphological rather than biological changes and may overestimate or underestimate residual disease due to treatment-induced fibrosis or necrosis^6^. Similarly, ultrasound (US) is operator-dependent and less sensitive in differentiating residual viable tumor from post-therapeutic tissue alterations^7^. These limitations highlight the need for imaging techniques that can provide complementary biological information beyond morphology. ^18^F-fluorodeoxyglucose (FDG) PET/CT is widely used to assess metabolic response in breast cancer^8^. By reflecting glucose metabolism, FDG PET/CT allows early detection of therapeutic response at the cellular level^9^. Nonetheless, FDG uptake may be nonspecific, influenced by inflammation or granulation tissue^10^, and its sensitivity is reduced in tumors with low metabolic activity, such as certain hormone receptor-positive/human epidermal growth factor receptor 2-negative (HR+/HER2−) subtypes or small residual foci after NAT^11^. Consequently, the diagnostic accuracy of FDG PET/CT for identifying complete or partial pathological responses remains suboptimal in the post-therapy setting.

^68^Ga-labeled fibroblast activation protein inhibitor (FAPI) PET/CT is an emerging imaging modality targeting cancer-associated fibroblasts (CAFs), a key component of the tumor microenvironment^12^. Unlike FDG, FAPI exhibits low background uptake and high tumor-to-background contrast, enabling clearer visualization of lesions even in low-metabolism cancers^13,14^. Moreover, FAPI uptake reflects stromal activation rather than glucose metabolism, potentially distinguishing viable tumor stroma from post-treatment inflammatory changes^12^. Preliminary studies have demonstrated the promising diagnostic performance of ^68^Ga-FAPI PET/CT in various malignancies, especially in breast cancer^15^.

However, few studies have directly compared the diagnostic performance of ^68^Ga-FAPI PET/CT and ^18^F-FDG PET/CT in evaluating treatment response after NAT, particularly regarding their ability to predict the pathological complete response (pCR) in primary breast lesions. Therefore, the present study aimed to compare the diagnostic accuracy of ^68^Ga-FAPI PET/CT and ^18^F-FDG PET/CT in assessing pathological response following NAT in patients with breast cancer.

## Results

### Patients

**Fig. 1:**
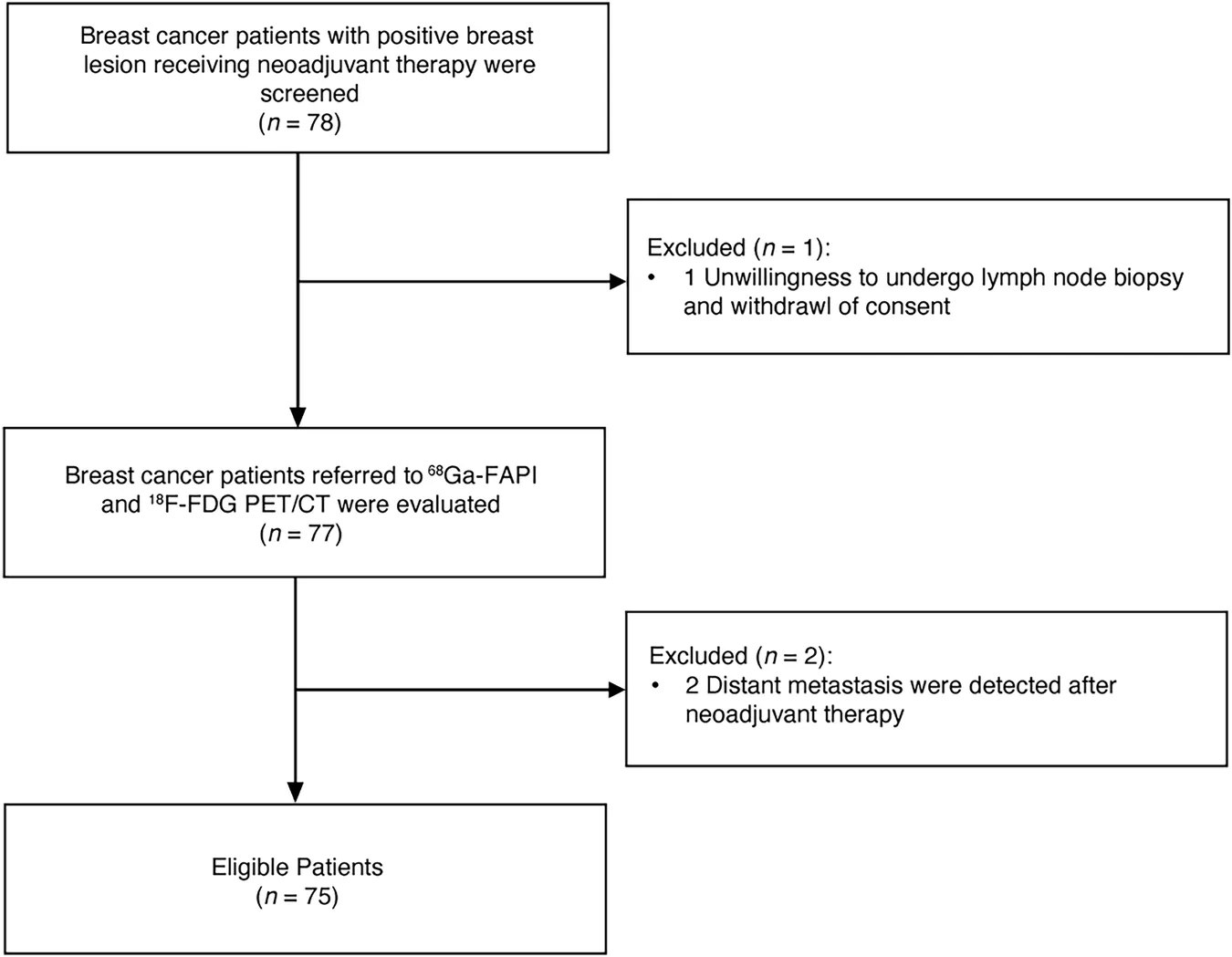
Flow chart of study enrollment. The diagram shows the process of patient screening, eligibility assessment, and final inclusion in the analysis. Reasons for exclusion are listed in the corresponding boxes, with the number of patients indicated.

A total of 78 patients was initially recruited between August 2024 and September 2025. Three patients were excluded: one unwillingness to undergo lymph node biopsy and withdrawl of consent, and two with newly detected distant metastases, resulting in 75 eligible participants (Fig. 1).

Among them, 33 (44.0%) achieved pCR of the primary tumor (ypT0/Tis), while 42 (56.0%) had residual invasive disease (ypT+). No significant differences were observed between the ypT0/Tis and ypT+ groups in terms of age, body mass index (BMI), menstrual status, clinical T/N stage, HER2 status, Ki-67 index, or pathologic subtype. Patients in the ypT0/Tis group were more likely to have estrogen receptor (ER) and progesterone receptor (PR)-negative tumors (81.8%, *p* < 0.001; and 78.8%, *p* = 0.003, respectively) and a higher proportion of HER2+ (54.6%) or triple-negative breast cancer (TNBC) (30.3%) subtypes (*p* < 0.001). Conversely, HR+/HER2− molecular subtype predominated in the ypT+ group (59.5%), followed by HER2+ (33.3%) and TNBC (7.1%). Detailed baseline characteristics are summarized in Table 1.

**Table 1:** Baseline characteristics and treatment

Characteristics	Whole population, *n* (%)	ypT+, *n* (%)	ypT0/Tis, *n* (%)	*P* value
*Age (years)*				*0.19*
<50	39 (52.0%)	19 (45.2%)	20 (60.6%)	
≥50	36 (48.0%)	23 (54.8%)	13 (39.4%)	
*BMI (kg/m*^*2*^)				*0.26*
<25	46 (61.3%)	26 (61.9%)	20 (60.6%)	
25–30	22 (29.3%)	14 (33.3%)	8 (24.2%)	
å 30	7 (9.3%)	2 (4.8%)	5 (15.2%)	
*Menstrual state*				*0.65*
Pre/perimenopausal	41 (54.7%)	22 (52.4%)	19 (57.6%)	
Postmenopausal	34 (45.3%)	20 (47.6%)	14 (42.4%)	
*cT stage*				*0.07*
T1	4 (5.3%)	0 (0.0%)	4 (12.1%)	
T2	57 (76.0%)	32 (76.2%)	25 (75.8%)	
T3	9 (12.0%)	7 (16.7%)	2 (6.1%)	
T4	5 (6.7%)	3 (7.1%)	2 (6.1%)	
*cN stage*				*0.48*
N1				
FNA (+)	10 (13.3%)	7 (16.7%)	3 (9.1%)	
FNA (−)*	2 (2.7%)	1 (2.4%)	1 (3.0%)	
N2	44 (58.7%)	26 (61.9%)	18 (54.5%)	
N3	19 (25.3%)	8 (19%)	11 (33.3%)	
*ER status*				***<0.001***
Negative	37 (49.3%)	10 (23.8%)	27 (81.8%)	
Positive	38 (50.7%)	32 (76.2%)	6 (18.2%)	
*PR status*				***0.003***
Negative	45 (60.0%)	19 (45.2%)	26 (78.8%)	
Positive	30 (40.0%)	23 (54.8%)	7 (21.2%)	
*HR status*				***<0.001***
Negative	35 (46.7%)	10 (23.8%)	25 (75.8%)	
Positive	40 (53.3%)	32 (76.2%)	8 (24.2%)	
*HER2 status*				*0.07*
Negative	43 (57.3%)	28 (66.7%)	15 (45.5%)	
Positive	32 (42.7%)	14 (33.3%)	18 (54.5%)	
*Ki-67*				*0.13*
≥14%	67 (89.3%)	35 (83.3%)	32 (97.0%)	
<14%	8 (10.7%)	7 (16.7%)	1 (3.0%)	
*Pathologic subtype*				*0.78*
IDC	70 (93.3%)	40 (95.2%)	30 (90.9%)	
Non-IDC	5 (6.7%)	2 (4.8%)	3 (9.1%)	
*Molecular subtype*				***<0.001***
HR+/HER2−	30 (40.0%)	25 (59.5%)	5 (15.2%)	
HER2+	32 (42.7%)	14 (33.3%)	18 (54.5%)	
TNBC	13 (17.3%)	3 (7.1%)	10 (30.3%)	

*ALND* axillary lymph node dissection, *BCS* breast conserving surgery, *BMI* body mass index, *ER* estrogen receptor, *FNA* fine-needle aspiration, *HER2* human epidermal growth factor receptor2, *HR* hormone receptor, *IDC* invasive ductal carcinoma, *PR* progesterone receptor, *TNBC* triple negative breast cancer.

*Two patients with clinical lymph node positivity but fine-needle aspiration negativity.

P values are italicized. Bold values are statistically signiﬁcant (P < 0.05).

### Diagnostic performance of ^18^F-FDG PET/CT in breast lesions after NAT

**Fig. 2:**
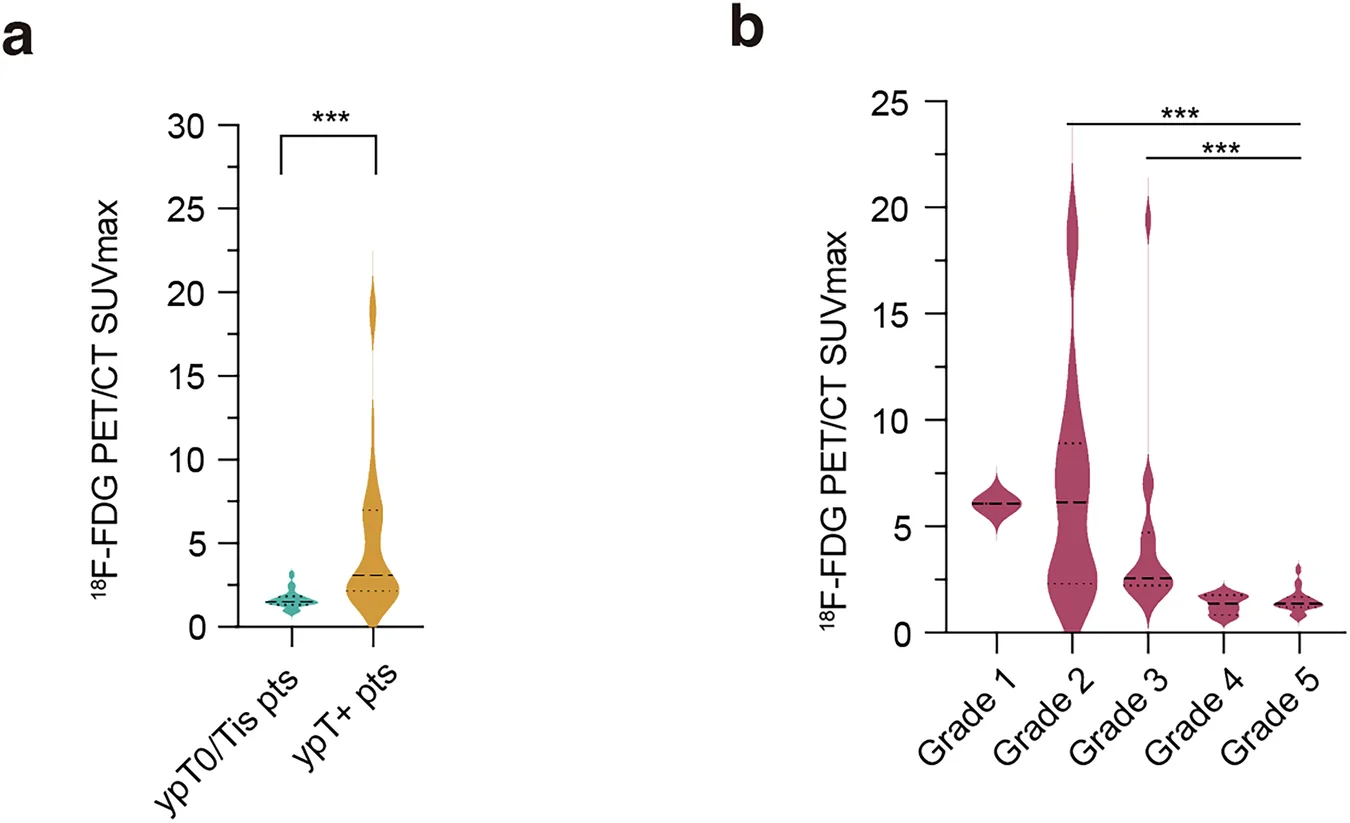
^18^F-FDG PET/CT parameters and ypT status after NAT in breast cancer. **a** ^18^F-FDG PET/CT PET/CT parameters uptake in breast tumor after NAT; **b** ^18^F-FDG PET/CT uptake according to Miller–Payne grades. *** represent *p* < 0.001 (student’s *t* test). NAT neoadjuvant therapy.

In the overall cohort, the maximum standardized uptake value (SUVmax) of ^18^F-FDG PET/CT was significantly higher in the ypT+ group than in the ypT0/Tis group (5.19 ± 4.68 vs. 1.45 ± 0.55, *p* < 0.001, Fig. 2a). High SUVmax values correctly identified 24 of 42 ypT+ patients (57.1%), whereas low SUVmax values correctly identified 31 of 33 ypT0/Tis patients (93.9%) (Table 2). These findings indicate that the SUVmax value of ^18^F-FDG PET/CT is not accurate for detecting residual disease after NAT. Regarding Miller–Payne (MP) grades after NAT (Fig. 2b), a negative correlation was noted between SUVmax and MP grades (*ρ* = −0.723, p < 0.001). SUVmax peaked in Grade 2 (central tendency: 6.70) and decreased dramatically from Grade 2 to Grade 4 (Grade 4 central tendency: 1.29). Grade 5 exhibited significantly lower SUVmax than Grade 2 (*p* < 0.001) and Grade 3 (*p* < 0.001), while no significant differences were observed between Grade 4 and Grade 5, indicating that patients with better pathological responses (higher grades) were generally associated with lower SUVmax distributions. To further evaluate its diagnostic potential, we performed receiver operating characteristic (ROC) curve analysis for ^18^F-FDG PET/CT, yielding an area under the curve (AUC) of 0.89. Based on standard and weighted Youden’s indices, two optimal operating points (OPs) were determined: OP1 at an SUVmax cutoff of 2.08 and OP2 at 3.11.

**Table 2:** Association between SUVmax (^18^F-FDG PET-CT and ^68^Ga-FAPI PET-CT) and ypT status in patients receiving NAT

SUVmax status*	ypT +	ypT0/Tis	Total
^*18*^*F-FDG PET-CT*			
High (*n*)	24	2	26
Low (*n*)	18	31	49
**Total**	42	33	75
^*68*^*Ga-FAPI PET-CT*			
High (*n*)	35	2	37
Low (*n*)	7	31	38
**Total**	42	33	75

*NAT* neoadjuvant therapy.

* The cutoff value of ^18^F-FDG PET/CT SUVmax was 2.5 for breast lesion and the cutoff value of ^68^Ga-FAPI PET/CT SUVmax was determined by Youden’s index (SUVmax 3.53 for breast lesion).

### Diagnostic performance of ^68^Ga-FAPI PET/CT in breast lesions after NAT

**Fig. 3:**
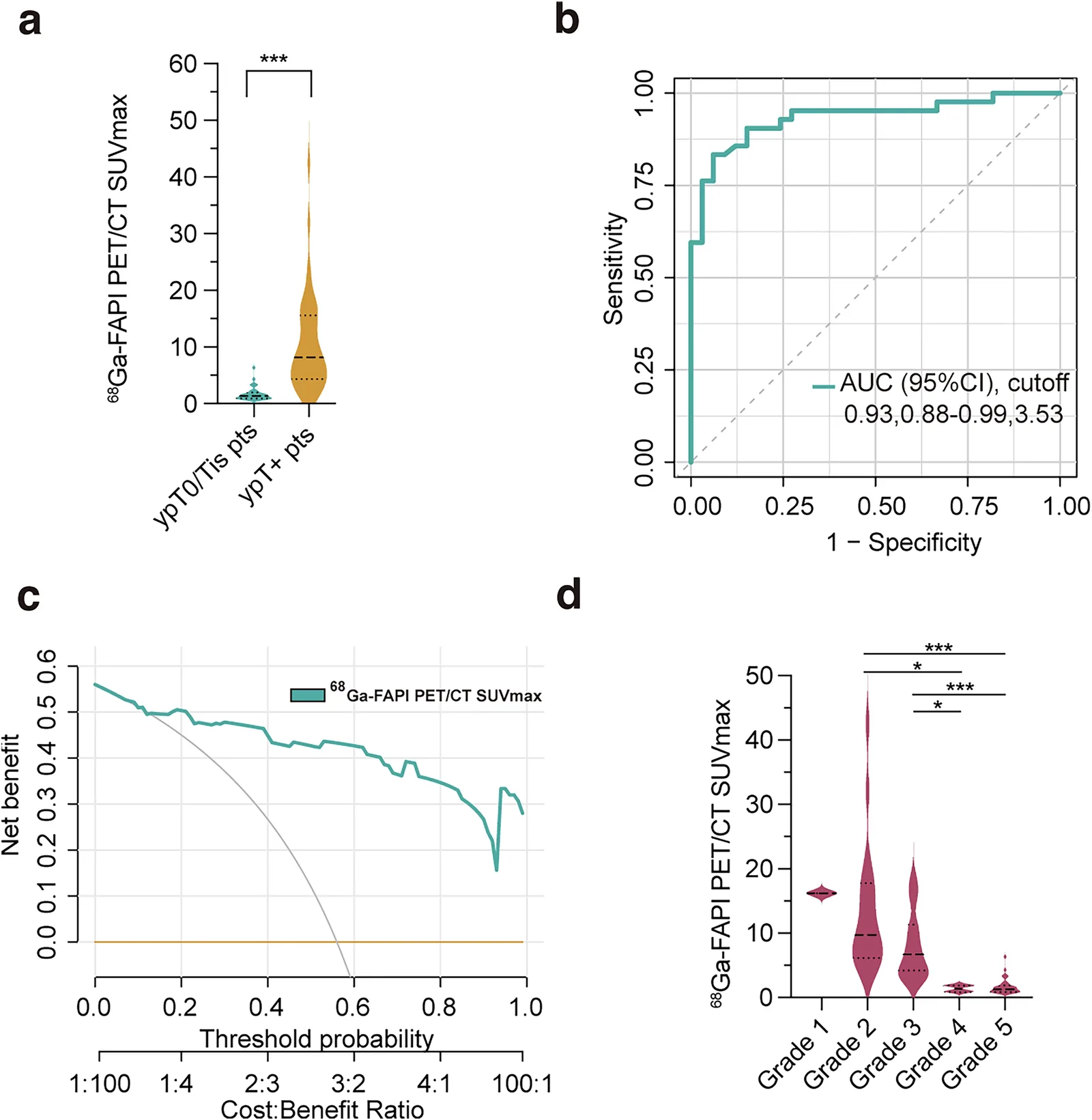
^68^Ga-FAPI PET/CT parameters and ypT status after NAT in breast cancer. **a** ^68^Ga-FAPI PET/CT parameters uptake in breast tumor after NAT; **b** AUC of ROC curve of breast uptake with ^68^Ga-FAPI PET/CT SUVmax; **c** Decision curve analysis of tumor uptake with ^68^Ga-FAPI PET/CT SUVmax; **d** ^68^Ga-FAPI PET/CT uptake according to Miller–Payne grades. *** represent *p* < 0.001, * represent *p* < 0.05 (student’s *t* test). NAT neoadjuvant therapy.

Similarly, ^68^Ga-FAPI PET/CT-derived SUVmax values were significantly higher in the ypT+ group than in the ypT0/Tis group (10.45 ± 8.54 vs. 1.59 ± 1.22, *p* < 0.001, Fig. 3a). ROC curve analysis was used to select optimal cutoff value of SUVmax for distinguishing ypT+ from ypT0/Tis lesions, with an AUC of 0.93 (Fig. 3b). Decision curve analysis validated its clinical utility, showing a net benefit for threshold probabilities above 0.1 (Fig. 3c). Using the Youden’s index-derived optimal cutoff of 3.53, high SUVmax values correctly identified 35 out of 42 ypT+ patients, with a sensitivity of 83.3%. Moreover, the low SUVmax values also correctly identified 31 out of 33 ypT0/Tis patients, with a specificity of 93.9% (Table 2), indicating that ^68^Ga-FAPI PET/CT was accurate for identifying pathological responses in NAT.

In addition, we quantified the distribution of SUVmax values according to MP grading categories (Fig. 3d). The highest FAPI uptake was observed in Grade 1 lesions (central tendency: 16.11), with SUVmax declining steadily from Grade 1 through Grade 4 (Grade 4 central tendency: 1.30), with a negative association between ^68^Ga-FAPI PET/CT SUVmax and MP grade (*ρ* = −0.809, *p* < 0.001).

### Comparison of ^18^F-FDG PET/CT and ^68^Ga-FAPI PET/CT in breast lesions after NAT

**Fig. 4:**
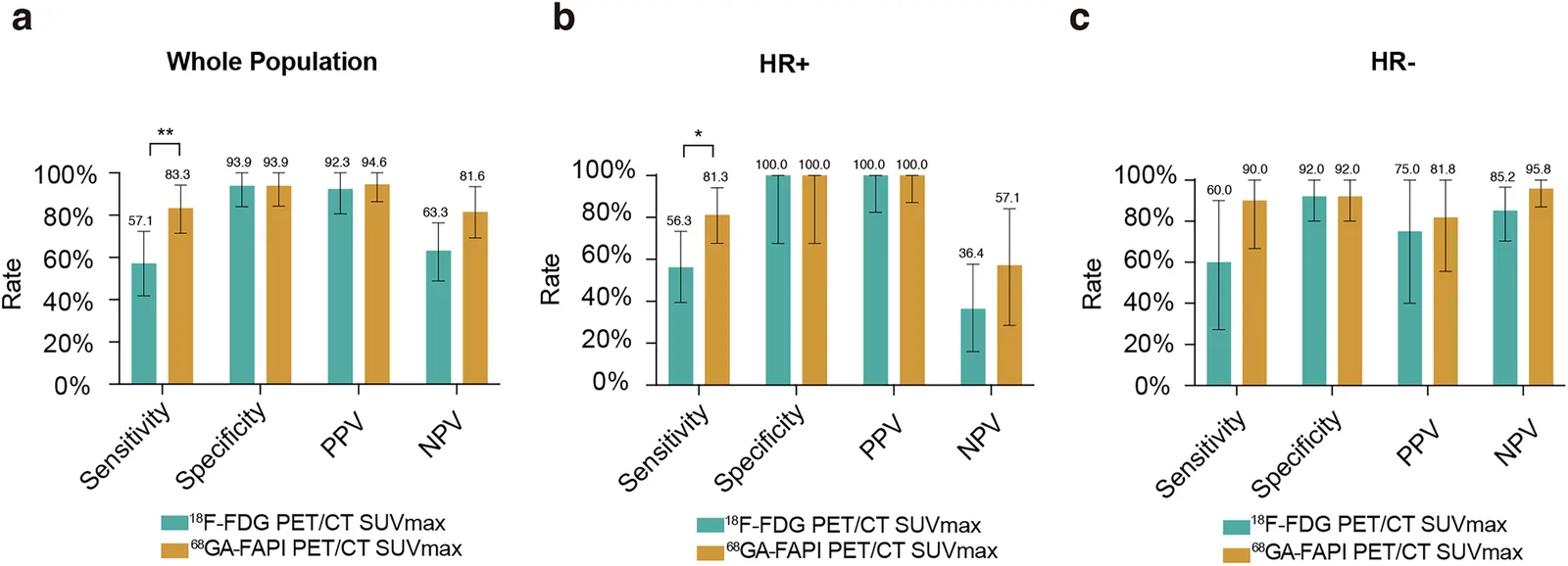
Comparison of diagnostic performances of ^18^F-FDG PET/CT and ^68^Ga-FAPI PET/CT in predicting ypT status. The diagnostic performances of ^18^F-FDG PET/CT and ^68^Ga-FAPI PET/CT are compared in **a** the whole population, **b** HR+, **c** HR−. The SUVmax has been recognized as predictor variable for ^18^F-FDG PET/CT ^68^Ga-FAPI PET/CT. Error bars represent 95% confidence intervals (CIs): percentiles from nonparametric bootstrap resampling were used for metrics with sufficient variability, and Wilson score intervals were applied when bootstrap resampling failed due to lack of variability. Statistical comparisons were performed using McNemar’s test (sensitivity/specificity) and Fisher’s exact test (PPV/NPV). ** represent *p* < 0.01. HR hormone receptor, NPV negative predictive value, PPV positive predictive value.

**Fig. 5:**
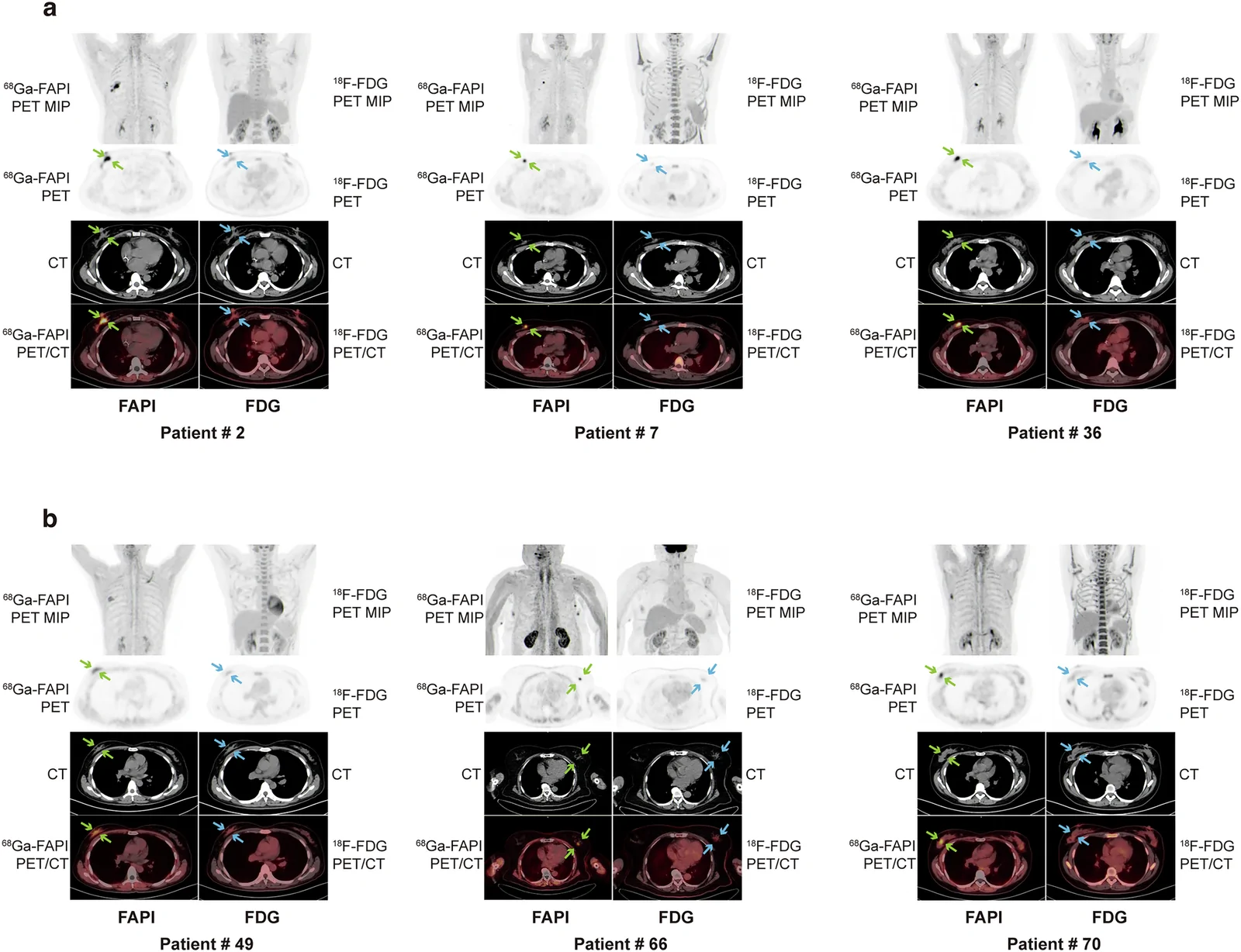
Representative cases of breast lesions showing with discrepant uptake on ^68^Ga-FAPI PET/CT and ^18^F-FDG PET/CT. **a** HR+/HER2− patients; **b** HER2+ patients. Green arrows indicate ^68^Ga-FAPI PET/CT images of breast lesion, and blue arrows indicate ^18^F-FDG PET/CT images of breast lesion.

In the overall cohort, ^68^Ga-FAPI PET/CT demonstrated significantly higher sensitivity than ^18^F-FDG PET/CT (83.3% vs. 57.1%, *p* = 0.003), and a similar trend was also observed in negative predictive value (NPV) (81.6% vs. 63.3%, *p* = 0.09). However, specificity and positive predictive value (PPV) were comparable between the two modalities (93.9% vs. 93.9%, *p* = 1.00; and 94.6% vs. 92.3%, *p* = 1.00, respectively) (Fig. 4a). Representative images of ^68^Ga-FAPI and ^18^F-FDG PET/CT from ypT+ patients further demonstrated the superior ability of ^68^Ga-FAPI PET/CT to detect residual breast lesions. Even when FDG uptake was faint, ^68^Ga-FAPI provided clear delineation of residual tumor (Fig. 5, Table S1).

Comparative ROC curve analysis revealed that ^68^Ga-FAPI PET/CT numerically outperformed ^18^F-FDG PET/CT, though the difference did not reach statistical significance (*p* = 0.09). The corresponding AUC values were 0.89 (95% CI: 0.81–0.97) for ^18^F-FDG PET/CT and 0.93 (95% CI: 0.88-0.99) for ^68^Ga-FAPI PET/CT (Figure S1a). Subsequent diagnostic performance evaluation was conducted using two data-driven optimal cut-off thresholds for ^18^F-FDG. When adopting the OP1 threshold (SUVmax cutoff = 2.08, 39 cases were ^18^F-FDG‑positive and 36 cases were ^18^F-FDG‑negative), ^18^F-FDG PET/CT yielded a sensitivity of 83.3%, specificity of 87.9%, PPV of 89.7% and NPV of 80.6%. No statistically significant differences were observed across all indices (83.3% vs. 83.3%, *p* = 1.00; 87.9% vs. 93.9%, *p* = 0.63; 89.7% vs. 94.6%, *p* = 0.68; 80.6% vs. 81.6%, *p* = 1.00) (Fig. S1b). When applying the OP2 threshold (SUVmax cutoff = 3.11, 21 cases were ^18^F‑FDG‑positive and 54 cases were ^18^F‑FDG‑negative), ^18^F‑FDG PET/CT achieved a sensitivity of 50.0%, specificity of 100.0%, PPV of 100.0%, and NPV of 61.1%. Compared with ^68^Ga‑FAPI PET/CT, ^18^F‑FDG PET/CT showed significantly lower sensitivity and NPV (50.0% vs. 83.3%, *p* < 0.001; 61.1% vs. 81.6%, *p* = 0.04), whereas specificity and PPV were comparable between the two modalities (100.0% vs. 93.9%, *p* = 0.50; 100.0% vs. 94.6%, *p* = 0.53) (Fig. S1c).

### Exploratory subgroup analysis according to HR status and molecular subtypes

The predictive performance of ^18^F-FDG and ^68^Ga-FAPI PET/CT was further evaluated across different HR statuses. In HR+ tumors, ^68^Ga-FAPI PET/CT demonstrated higher sensitivity (81.3% vs. 56.3%, *p* = 0.02) than ^18^F-FDG PET/CT. Both modalities had low to moderate NPV (57.1% vs. 36.4%, *p* = 0.31). Specificity and PPV were comparable between the two modalities (100.0% vs. 100.0%, *p* = 1.00; and 100.0% vs. 100.0%, *p* = 1.00, respectively). In HR− tumors, ^68^Ga-FAPI PET/CT also showed a trend toward higher sensitivity and NPV compared with ^18^F-FDG PET/CT (90.0% vs. 60.0%, *p* = 0.25; and 95.8% vs. 85.2%, *p* = 0.35, respectively) (Fig. 4).

Regarding molecular subtypes, ^68^Ga-FAPI PET/CT tended to have higher sensitivity than ^18^F-FDG PET/CT in HR+/HER2− (80.0% vs. 56.0%, *p* = 0.07) and HER2+ tumors (92.9% vs. 57.1%, *p* = 0.06), though these differences did not reach statistical significance, while specificity was consistently high and comparable between the two modalities in these two subtypes (HR+/HER2−: both 100.0%, *p* = 1.00; HER2+: 88.9% vs. 94.4%, *p* = 1.00); in TNBC, sensitivity (66.7% vs. 66.7%, *p* = 1.00) and specificity (100.0% vs. 90.0%, *p* = 1.00) were similar for both imaging techniques (Fig. S2). Further subgroup analysis stratified by pembrolizumab administration showed comparable diagnostic performance between the two modalities (Tables S2 and S3, Fig. S3).

## Discussion

In this study, ^68^Ga-FAPI PET/CT demonstrated superior sensitivity over ^18^F-FDG PET/CT in evaluating breast pathological response after NAT, while maintaining high specificity, supporting ^68^Ga-FAPI PET/CT was a more reliable imaging tool to predict residual disease after NAT and guide further personalized treatment decisions. Accurate assessment of residual disease after NAT is essential for determining whether patients should proceed to surgery or continue systemic therapy. However, conventional imaging methods, including MRI and ^18^F‑FDG PET/CT, often struggle to differentiate residual tumor from post-treatment fibrosis or necrosis^6,10^, leading to inaccurate differentiation between responders and non-responders. The reported sensitivity of ultrasound in predicting pCR in breast cancer patients was only 36.2%^16^, while the sensitivity of MRI ranges widely from 44.4% to 83% in different studies^16–19^, highlighting the need for more reliable tools. Previous studies have assessed the predictive value of ^18^F‑FDG or ^68^Ga‑FAPI PET/CT alone in breast cancer undergoing NAT^20–22^. Here, we present the first prospective direct comparison of ^18^F‑FDG and ^68^Ga‑FAPI PET/CT for evaluating NAT response in breast cancer. Of note, we used a clinically validated fixed threshold for ^18^F‑FDG, while a data-driven threshold was applied for ^68^Ga‑FAPI due to the absence of an established consensus cutoff in this clinical setting. We found that ^68^Ga-FAPI PET/CT demonstrated markedly superior sensitivity compared with ^18^F-FDG PET/CT for detecting residual invasive breast cancer, while maintaining high and comparable specificity and PPV. In subgroup analysis, ^68^Ga-FAPI PET/CT demonstrated higher sensitivity in HR+ breast tumors. To mitigate potential bias introduced by differing threshold strategies, we additionally performed ROC curve analysis for ^18^F-FDG and conducted comparative assessments using ROC-derived cutoffs. Nevertheless, our study enrolled a relatively limited number of participants. In addition, the determination of ^18^F-FDG cutoff values is objectively influenced by different NAT cycles and therapeutic regimens^23–25^. Thus, the most appropriate SUVmax threshold dedicated to ^18^F-FDG PET/CT for breast cancer NAT response evaluation still requires further verification in larger-scale clinical studies. Early and accurate recognition of non-responders enables timely treatment escalation strategies, such as extending NAT duration, modifying regimens, introducing novel agents, or enrolling patients in clinical trials, so as to avoid prolonging ineffective treatment. A practical advantage of ^68^Ga-FAPI PET/CT is that it does not require fasting^26^, simplifying preparation and enhancing operational convenience. Accurate identification of responders is also clinically meaningful, including reduced treatment intensity, shortened therapy duration, or even surgical de-escalation. With growing interest in avoiding breast surgery for patients achieving pCR^27^, ^68^Ga-FAPI PET/CT could enable noninvasive identification of complete responders, and inform surgical de-escalation strategies.

The superior performance of ^68^Ga-FAPI PET/CT might result from its distinct biological targeting mechanism. While FDG reflects tumor glucose metabolism^9^, its sensitivity can be limited by factors such as ‘metabolic stunning’ of residual viable tumor tissue after NAT^28^. In contrast, FAPI binds to fibroblast activation protein expressed on CAFs within the tumor stroma^12^. CAFs are abundant in the breast cancer microenvironment and persist in areas of drug-resistant tumor or treatment-induced fibrosis^28^. Because CAFs are closely associated with the tumor or its reactive stroma, FAPI PET/CT indirectly indicates residual tumor tissue, highlighting regions that FDG may miss^29,30^. HR+ breast cancer generally show lower glucose metabolic activity than TNBC or HER2+ breast cancer^11^. This provides a mechanistic explanation for the superior accuracy of ^68^Ga-FAPI in this subtype. Furthermore, immune checkpoint inhibitors may induce atypical response patterns that can confound imaging-based assessment^31^. We therefore performed subgroup analysis by pembrolizumab administration, which demonstrated that the comparative performance of ^68^Ga‑FAPI and ^18^F‑FDG PET/CT was not significantly influenced by immunotherapy.

This study has several limitations. First, it was a single-center prospective observational study with a relatively small sample size, which may limit the generalizability of the findings, especially for certain subgroup analysis. Second, in our study, the optimal SUVmax cutoff for ^68^Ga‑FAPI was determined by Youden’s index, whereas a fixed threshold was adopted for ^18^F‑FDG, future studies are needed to define the optimal SUVmax cutoff for ^68^Ga‑FAPI. Third, the present study only focused on the primary breast tumor without evaluating axillary lymph node status, and lacked a direct comparison of diagnostic performance between ^68^Ga-FAPI PET/CT and breast MRI. These issues warrant further investigation in future studies. Multicenter studies with larger cohorts are warranted to validate these findings and to further improve the predictive accuracy of ^68^Ga-FAPI PET/CT in assessing NAT response in patients with breast cancer.

In conclusion, ^68^Ga-FAPI PET/CT demonstrated superior sensitivity compared with ^18^F-FDG PET/CT for evaluating residual disease in primary breast tumors of clinical axillary lymph node positivity (cN+) breast cancer patients after neoadjuvant therapy. ^68^Ga-FAPI shows promise as a valuable imaging tool to guide personalized treatment during NAT and surgical decision making.

## Methods

### Study design and patient population

This single-center phase II prospective observational cohort study was approved by the Ethics Committee of Ruijin Hospital, Shanghai Jiao Tong University School of Medicine (Approval No. 2024174) and registered at ClinicalTrials.gov (NCT06559371). The study was conducted in accordance with the ethical standards of the Declaration of Helsinki and its later amendments. From August 2024 to September 2025, patients with newly diagnosed, pathologically confirmed invasive breast cancer who received NAT followed by surgery were consecutively enrolled. All participants provided written informed consent prior to any study-related procedures, including ^68^Ga-FAPI PET/CT and ^18^F-FDG PET/CT examinations.

The eligibility criteria were as follows: (1) female patients aged ≥18 years; (2) Eastern Cooperative Oncology Group (ECOG) performance status of 0–2; (3) pathological confirmation of malignant breast tumor; (4) cN+; (5) completion of at least three cycles of NAT before surgery; and (6) the availability of both ^68^Ga-FAPI and ^18^F-FDG PET/CT scans performed within 3 weeks after NAT and before definitive surgery. Patients with distant metastasis, incomplete imaging data, missing pathological confirmation after surgery, or refusal to complete the required axillary lymph node biopsy were excluded.

### NAT treatment

NAT regimens were selected according to molecular subtype and contemporary clinical guidelines.

For HR + /HER2- breast cancer, patients generally received four 21-day cycles of epirubicin (90 mg/m²) plus cyclophosphamide (600 mg/m²) (EC regimen), followed by 4 cycles of docetaxel (80–100 mg/m²) administered every 21 days (EC-T)^32^.

For HER2-positive tumors, most patients were treated with trastuzumab and pertuzumab in combination with taxane- and platinum-based chemotherapy. Specifically, trastuzumab was given either intraveneously at an initial dose of 8 mg/kg followed by 6 mg/kg every 21 days, or subcutaneously at 600 mg every 21 days, and pertuzumab at 840 mg initially followed by 420 mg every 21 days for 4–6 cycles. Docetaxel (75 mg/m^2^ d1, cycled every 21 days for 6 cycles) and carboplatin (at a dose based on an area under the concentration-time curve of 5–6 mg/ml/min d1, cycled every 21 days for 6 cycles) were co-administered^33^. One patient received a regimen consisting of EC-T + HP, and four patients received pyrotinib-containing regimens (320 mg d1–d21, cycled every 21 days for 2 cycles).

For TNBC, the typical regimen included weekly paclitaxel (80 mg/m²) or nab-paclitaxel (100 mg/m²) together with carboplatin (at a dose based on an area under the concentration-time curve of 1.5 mg/ml/min) on days 1, 8, and 15 of a 28-day cycle, followed by four cycles of EC. Additionally, ten TNBC patients received pembrolizumab (200 mg) as part of combination NAT^34^.

### Imaging protocols

^68^Ga-FAPI PET/CT: Patients were administered an intravenous dose of ^68^Ga-FAPI ranging from 1.1 to 1.54 mCi (40.7–56.98 MBq). PET/CT imaging was performed at Ruijin Hospital using the uEXPLORER scanner (United Imaging, China). Each scan was acquired from the head to the feet with a standardized acquisition time of 5 min^35,36^. PET data were reconstructed with the Ordered Subsets Expectation Maximization (OSEM) algorithm, employing 2 iterations and 20 subsets, resulting in a voxel dimension of 3.125 × 3.125 × 2.886 mm³. CT acquisition was conducted with a tube current of 180 mA, voltage of 120 kV, and slice thickness of 3 mm. PET/CT scans were acquired approximately 60 min post-injection^13,37,38^.

^18^F-FDG PET/CT: Patients were fasted for ≥6 h prior to the examination and rested for 60–90 min after intraveneous injection of ^18^F-FDG (3.7–4.44 MBq/kg)^39,40^. PET/CT scans were performed at Ruijin Hospital from the base of the head to the upper thigh or from the top of the head to the foot using hybrid PET/CT scanners (Discovery MI, GE Healthcare; uEXPLORER, UIH). Each scan was acquired from the head to the feet using the uEXPLORER scanner with a standardized acquisition time of 3 min^41^. For Discovery MI, GE Healthcare, the following CT parameters were used: tube voltage of 120 kV, automatic mA-modulation, and slice thickness of 3.75 mm. PET images were obtained in 3D mode and reconstructed into a 256 × 256 matrix size by using either the Bayesian penalised likelihood reconstruction algorithm (Q.clear; GE Healthcare), with a penalisation factor (*β*) of 500, or the OSEM. For 2. uEXPLORER, UIH, the following CT parameters were used: tube voltage of 120 kV, automatic mA-modulation, and slice thickness of 3 mm. PET images were obtained in 3D mode and reconstructed in a 256 × 256 matrix size (iteration: 2, subset: 20) by using OSEM PET.

All image analyses were independently performed by two nuclear medicine physicians. For primary breast lesions, three-dimensional volumes of interest (VOI) were delineated, and a semi-automatic segmentation was applied using a threshold of 40% of the SUVmax to define the lesion boundary, from which the corresponding SUVmax value was obtained.

### Clinical and pathological assessment

Before initiating NAT, clinical tumor (cT) and nodal (cN) stages were determined through physical examination and breast ultrasonography, in accordance with the 8^th^ edition of the American Joint Committee on Cancer (AJCC) Staging Manual^42^. All patients were diagnosed with breast cancer based on ultrasound-guided core needle biopsy prior to treatment. After surgery, breast specimens, sentinel lymph nodes (SLNs), and axillary lymph nodes (ALNs) were examined. Pathological and immunohistochemistry (IHC) evaluations were independently performed by at least two experienced pathologists, following standardized procedures and American Society of Clinical Oncology/College of American Pathologists (ASCO/CAP) guidelines.

Pathological response to NAT was evaluated using the MP grading system as previously described^43^. According to the 8th edition of the AJCC Staging Manual^42^, patients were further categorized into ypT0/Tis (MP grade 5) and ypT+ (MP grades 1–4) for diagnostic performance analysis.

HR positivity was defined as ≥1% nuclear staining for ER and/or PR in invasive tumor cells^44^. HER2 positivity was determined by an IHC score of 3+ or confirmed amplification by fluorescence in situ hybridization (FISH)^45^. TNBC was characterized by the absence of ER, PR, and HER2 expression. The Ki-67 proliferation index was assessed using the standardized scoring method recommended by the International Ki-67 Working Group^46^.

### Statistical analysis

All statistical analyses were performed using R software (version 4.3.1), and figures were generated with GraphPad Prism (version 10.4.2). Continuous variables were expressed as mean ± standard deviation (SD), and categorical variables were presented as counts and percentages. Comparisons between the ypT0/Tis and the ypT+ groups were conducted using Student’s *t*-test for continuous variables, and chi-square or Fisher’s exact test for categorical variables, as appropriate. For paired binary outcomes, such as sensitivity and specificity between ^68^Ga-FAPI and ^18^F-FDG PET/CT, McNemar’s test (exact binomial test for small discordant counts) was used to evaluate differences. DeLong’s test was applied to compare the AUCs of ROC curves between ^18^F-FDG PET/CT and ^68^Ga-FAPI PET/CT.

The diagnostic performance of ^68^Ga-FAPI PET/CT and ^18^F-FDG PET/CT in detecting residual breast tumors after NAT was assessed by calculating sensitivity, specificity, PPV, and NPV. For ^18^F-FDG PET/CT, a fixed SUVmax cutoff of 2.5 was adopted in accordance with widely accepted clinical guidelines and research standards^47–50^. Consistently, several prior studies have utilized an SUVmax cutoff of 2.5 for ^18^F-FDG PET/CT to evaluate NAT response in breast cancer^51–53^. To comprehensively assess its performance, ROC curve analysis was also performed for ^18^F-FDG PET/CT. Guided by relevant literature^54,55^, two OPs were identified: OP1 was derived using the standard Youden’s index to maximize (sensitivity + specificity − 1), while OP2 employed a weighted Youden’s index to maximize [2 × (1/4 × sensitivity + 3/4 × specificity) − 1], prioritizing higher specificity to minimize misdiagnosis risk. Given the lack of a validated consensus threshold for ^68^Ga-FAPI PET/CT in this setting, its optimal cutoff was determined via ROC curve analysis and Youden’s index based on the entire study population. Subgroup analyses were performed according to HR status and molecular subtype (HR+/HER2−, HER2+, TNBC). In TNBC patients, further subgroup analysis was performed according to whether pembrolizumab was administered or not.

## Supplementary information


Supplementary materials_clean version.


## Data Availability

The datasets analyzed during the current study are not publicly available as participants of this study did not agree for their data to be shared publicly. However, data can be made available from the corresponding author on reasonable request under a data transfer agreement and upon Ethics Committee approval.

## Code availability

No custom code was used in this study.
