# Supplementary material for: Diagnostic Performance of 68Ga-FAPI PET/CT Versus 18F-FDG PET/CT in Evaluating Neoadjuvant Therapy Pathological Response in Breast Cancer
Source: NPJ Breast Cancer. 2026 Jun 2;12:115. doi: 10.1038/s41523-026-00983-4 (PMC13534706; doi:10.1038/s41523-026-00983-4)
Supplement: Supplementary file 1 [file 41523_2026_983_MOESM1_ESM.docx]

**Supplementary materials**

1. Table S1. Detailed clinical information of enrolled patients and SUVmax values of breast lesions on ^18^F-FDG PET/CT and ^68^Ga-FAPI PET/CT.
2. Table S2. Association between SUVmax (^18^F-FDG PET-CT and ^68^Ga-FAPI PET-CT) and ypT status in TNBC patients receiving NAT with pembrolizumab
3. Table S3. Association between SUVmax (^18^F-FDG PET-CT and ^68^Ga-FAPI PET-CT) and ypT status in TNBC patients receiving NAT without pembrolizumab
4. Figure S1. ROC curves and diagnostic performance of ^18^F-FDG and ^68^Ga-FAPI PET/CT for predicting ypT status in breast cancer.
5. Figure S2. Comparison of diagnostic performances of ^18^F-FDG PET/CT and ^68^Ga-FAPI PET/CT in predicting ypT status for (a) HR+/HER2-, (b) HER2+, (c) TNBC population after NAT.
6. Figure S3. Comparison of diagnostic performances of ^18^F-FDG PET/CT and ^68^Ga-FAPI PET/CT in predicting ypT status after NAT for (a) TNBC treated with pembrolizumab, and (b) TNBC treated without pembrolizumab.
7. STROBE checklist

| **Table S1. Detailed clinical information of enrolled patients and SUVmax values of breast lesions on ^18^F-FDG PET/CT and ^68^Ga-FAPI PET/CT.** | | | | | | | | | |
| --- | --- | --- | --- | --- | --- | --- | --- | --- | --- |
| **FAPI No.** | **Age** | **pre-cT** | **Subtype** | **^18^F-FDG PET/CT SUVmax** | **^18^F-FDG PET/CT Status** | **^68^Ga-FAPI PET/CT SUVmax** | **^68^Ga-FAPI PET/CT SUVmax** | **ypT** |  |
| 1 | 36 | 2 | HER2+ | 1.46 | low | 1.46 | low | 0/is |  |
| 2 | 50 | 2 | HR+/HER2- | 2.11 | low | 11.07 | high | 2 |  |
| 3 | 35 | 2 | HER2+ | 1.24 | low | 1.51 | low | 0/is |  |
| 4 | 63 | 2 | HER2+ | 2.23 | low | 1.92 | low | 0/is |  |
| 5 | 45 | 2 | HR+/HER2- | 2.99 | high | 3.28 | low | 2 |  |
| 6 | 52 | 2 | HR+/HER2- | 1.12 | low | 0.74 | low | 1 |  |
| 7 | 44 | 2 | HR+/HER2- | 1.22 | low | 9.59 | high | 1 |  |
| 8 | 61 | 2 | HER2+ | 3.03 | high | 3.28 | low | 0/is |  |
| 9 | 36 | 4 | TNBC | 2.81 | high | 3.29 | low | 0/is |  |
| 10 | 71 | 4 | HER2+ | 1.16 | low | 0.67 | low | 0/is |  |
| 11 | 49 | 2 | HR+/HER2- | 2.39 | low | 6.59 | high | 2 |  |
| 12 | 40 | 2 | HER2+ | 2.39 | low | 4.19 | high | 0/is |  |
| 13 | 31 | 2 | HR+/HER2- | 2.18 | low | 4.4 | high | 2 |  |
| 14 | 51 | 3 | HER2+ | 2.59 | high | 3.76 | high | 1 |  |
| 15 | 41 | 2 | HER2+ | 2.06 | low | 6.21 | high | 0/is |  |
| 16 | 60 | 2 | HR+/HER2- | 2.52 | high | 5.02 | high | 2 |  |
| 17 | 38 | 2 | HER2+ | 0.75 | low | 0.51 | low | 0/is |  |
| 18 | 62 | 2 | HR+/HER2- | 2.47 | low | 5.73 | high | 2 |  |
| 19 | 48 | 3 | HER2+ | 1.21 | low | 2.11 | low | 0/is |  |
| 20 | 37 | 2 | TNBC | 3.73 | high | 5.92 | high | 1 |  |
| 21 | 72 | 2 | TNBC | 1.14 | low | 1.51 | low | 0/is |  |
| 22 | 68 | 2 | HR+/HER2- | 3.19 | high | 7.53 | high | 2 |  |
| 23 | 33 | 2 | HER2+ | 1.18 | low | 1.53 | low | 0/is |  |
| 24 | 45 | 1 | TNBC | 1.29 | low | 0.66 | low | 0/is |  |
| 25 | 51 | 2 | HER2+ | 6.63 | high | 8.13 | high | 2 |  |
| 26 | 35 | 2 | HER2+ | 1.61 | low | 2.99 | low | 0/is |  |
| 27 | 57 | 2 | HR+/HER2- | 8.48 | high | 8.26 | high | 2 |  |
| 28 | 53 | 2 | HER2+ | 0.82 | low | 0.76 | low | 0/is |  |
| 29 | 51 | 2 | HR+/HER2- | 0.68 | low | 0.91 | low | 1 |  |
| 30 | 25 | 1 | HR+/HER2- | 1.45 | low | 0.85 | low | 0/is |  |
| 31 | 60 | 2 | HER2+ | 18.02 | high | 8.31 | high | 2 |  |
| 32 | 55 | 1 | HR+/HER2- | 0.73 | low | 0.49 | low | 0/is |  |
| 33 | 32 | 2 | HR+/HER2- | 0.6 | low | 0.85 | low | 0/is |  |
| 34 | 54 | 3 | HER2+ | 1.36 | low | 1.76 | low | 0/is |  |
| 35 | 57 | 4 | HER2+ | 2.27 | low | 3.97 | high | 2 |  |
| 36 | 34 | 2 | HR+/HER2- | 2.23 | low | 11.28 | high | 1 |  |
| 37 | 51 | 4 | HR+/HER2- | 3.9 | high | 8.54 | high | 2 |  |
| 38 | 43 | 3 | HER2+ | 6.75 | high | 10.55 | high | 3 |  |
| 39 | 74 | 3 | HER2+ | 2.32 | low | 4.06 | high | 1 |  |
| 40 | 53 | 2 | HER2+ | 1.69 | low | 1.08 | low | 0/is |  |
| 41 | 38 | 2 | HR+/HER2- | 8.84 | high | 13.41 | high | 2 |  |
| 42 | 35 | 2 | HR+/HER2- | 4.28 | high | 13.59 | high | 2 |  |
| 43 | 31 | 2 | HR+/HER2- | 5.24 | high | 13.66 | high | 2 |  |
| 44 | 55 | 2 | HER2+ | 7.15 | high | 15.37 | high | 2 |  |
| 45 | 50 | 2 | TNBC | 1.27 | low | 1.04 | low | 0/is |  |
| 46 | 61 | 2 | HR+/HER2- | 5.88 | high | 16.11 | high | 2 |  |
| 47 | 47 | 3 | HR+/HER2- | 10.03 | high | 16.18 | high | 2 |  |
| 48 | 50 | 2 | TNBC | 1.27 | low | 1.04 | low | 0/is |  |
| 49 | 41 | 2 | HER2+ | 1.91 | low | 4.4 | high | 2 |  |
| 50 | 35 | 2 | HR+/HER2- | 2.09 | low | 2.79 | low | 1 |  |
| 51 | 72 | 1 | HR+/HER2- | 0.89 | low | 0.83 | low | 0/is |  |
| 52 | 33 | 3 | HR+/HER2- | 19.36 | high | 16.82 | high | 2 |  |
| 53 | 39 | 2 | TNBC | 1.32 | low | 0.75 | low | 0/is |  |
| 54 | 44 | 2 | HER2+ | 1.58 | low | 1.75 | low | 1 |  |
| 55 | 55 | 3 | HER2+ | 6.08 | high | 17.63 | high | 3 |  |
| 56 | 49 | 2 | TNBC | 12.15 | high | 18.02 | high | 2 |  |
| 57 | 36 | 2 | HR+/HER2- | 4.82 | high | 18.56 | high | 2 |  |
| 58 | 51 | 2 | HR+/HER2- | 2.13 | low | 6.03 | high | 2 |  |
| 59 | 40 | 2 | TNBC | 1.85 | low | 1.24 | low | 0/is |  |
| 60 | 70 | 3 | TNBC | 1.76 | low | 1.81 | low | 1 |  |
| 61 | 34 | 2 | TNBC | 1.4 | low | 1.3 | low | 0/is |  |
| 62 | 53 | 2 | HER2+ | 1.51 | low | 2.23 | low | 0/is |  |
| 63 | 76 | 2 | HER2+ | 6.97 | high | 21.23 | high | 2 |  |
| 64 | 69 | 2 | HR+/HER2- | 7.06 | high | 23.79 | high | 1 |  |
| 65 | 45 | 2 | TNBC | 1.48 | low | 1.54 | low | 0/is |  |
| 66 | 63 | 4 | HER2+ | 1.32 | low | 7.11 | high | 1 |  |
| 67 | 50 | 2 | HER2+ | 19.02 | high | 32 | high | 2 |  |
| 68 | 46 | 2 | HER2+ | 1.68 | low | 1.17 | low | 0/is |  |
| 69 | 31 | 2 | HER2+ | 1.31 | low | 1.45 | low | 0/is |  |
| 70 | 42 | 2 | HER2+ | 2.29 | low | 6 | high | 1 |  |
| 71 | 25 | 2 | TNBC | 1.53 | low | 0.62 | low | 0/is |  |
| 72 | 36 | 2 | HR+/HER2- | 7.67 | high | 42.46 | high | 2 |  |
| 73 | 48 | 2 | HR+/HER2- | 1.07 | low | 0.62 | low | 0/is |  |
| 74 | 56 | 2 | HER2+ | 1.11 | low | 0.97 | low | 0/is |  |
| 75 | 56 | 2 | HR+/HER2- | 2.45 | low | 2.72 | low | 1 |  |
| Note: Abbreviation: HER2, human epidermal growth factor receptor2; HR, hormone receptor; TNBC, triple negative breast cancer. | | | | | | | | | |

| **Table S2. Association between SUVmax (^18^F-FDG PET-CT and ^68^Ga-FAPI PET-CT) and ypT status in TNBC patients receiving NAT with pembrolizumab** | | | |
| --- | --- | --- | --- |
| **SUVmax status*** | **ypT+** | **ypT0/Tis** | **Total** |
| **^18^F-FDG PET-CT** |  |  |  |
| High (n) | 2 | 1 | 3 |
| Low (n) | 0 | 7 | 7 |
| **Total** | 2 | 8 | 10 |
| **^68^Ga-FAPI PET-CT** |  |  |  |
| High (n) | 2 | 0 | 2 |
| Low (n) | 0 | 8 | 8 |
| **Total** | 2 | 8 | 10 |
| Note: Abbreviation: TNBC, triple negative breast cancer; NAT, neoadjuvant therapy. *The cutoff value of ^18^F-FDG PET/CT SUVmax was 2.5 for breast lesion) and the cutoff value of ^68^Ga-FAPI PET/CT SUVmax was determined by Youden’s index (SUVmax 3.53 for breast lesion). | | | |

| **Table S3. Association between SUVmax (^18^F-FDG PET-CT and ^68^Ga-FAPI PET-CT) and ypT status in TNBC patients receiving NAT without pembrolizumab** | | | |
| --- | --- | --- | --- |
| **SUVmax status*** | **ypT+** | **ypT0/Tis** | **Total** |
| **^18^F-FDG PET-CT** |  |  |  |
| High (n) | 0 | 0 | 0 |
| Low (n) | 1 | 2 | 3 |
| **Total** | 1 | 2 | 3 |
| **^68^Ga-FAPI PET-CT** |  |  |  |
| High (n) | 0 | 0 | 0 |
| Low (n) | 1 | 2 | 3 |
| **Total** | 1 | 2 | 3 |
| Note: Abbreviation: TNBC, triple negative breast cancer; NAT, neoadjuvant therapy. * The cutoff value of ^18^F-FDG PET/CT SUVmax was 2.5 for breast lesion) and the cutoff value of ^68^Ga-FAPI PET/CT SUVmax was determined by Youden’s index (SUVmax 3.53 for breast lesion). | | | |

**Figure S1.** **ROC curves and diagnostic performance of ^18^F-FDG and ^68^Ga-FAPI PET/CT for predicting ypT status in breast cancer. (a) Comparative ROC curves for ^18^F-FDG PET/CT (cyan) and ^68^Ga-FAPI PET/CT (yellow); (b) Overall diagnostic performance using the OP1 cutoff; (c) Overall diagnostic performance using the OP2 cutoff.**

The SUVmax has been recognized as predictor variable for ^18^F-FDG PET/CT and ^68^Ga-FAPI PET/CT. Error bars represent 95% confidence intervals (CIs): percentiles from nonparametric bootstrap resampling were used for metrics with sufficient variability, and Wilson score intervals were applied when bootstrap resampling failed due to lack of variability.

Statistical comparisons were performed using McNemar’s test (sensitivity/specificity) and Fisher’s exact test (PPV/NPV).

*** represent p < 0.001, * represent p < 0.05. NPV, negative predictive value; PPV, positive predictive value.

**Figure S2. Comparison of diagnostic performances of ^18^F-FDG PET/CT and ^68^Ga-FAPI PET/CT in predicting ypT status for (a) HR+/HER2-, (b) HER2+, (c) TNBC population after NAT.** The SUVmax has been recognized as predictor variable for ^18^F-FDG PET/CT ^68^Ga-FAPI PET/CT. Error bars represent 95% confidence intervals (CIs): percentiles from nonparametric bootstrap resampling were used for metrics with sufficient variability, and Wilson score intervals were applied when bootstrap resampling failed due to lack of variability. Statistical comparisons were performed using McNemar’s test (sensitivity/specificity) and Fisher’s exact test (PPV/NPV).

HR, hormone receptor; NAT, neoadjuvant therapy; NPV, negative predictive value; PPV, positive predictive value; TNBC, triple negative breast cancer.

**Figure S3. Comparison of diagnostic performances of ^18^F-FDG PET/CT and ^68^Ga-FAPI PET/CT in predicting ypT status after NAT for (a) TNBC treated with pembrolizumab, and (b) TNBC treated without pembrolizumab.** The SUVmax has been recognized as predictor variable for ^18^F-FDG PET/CT ^68^Ga-FAPI PET/CT. Error bars represent 95% confidence intervals (CIs): percentiles from nonparametric bootstrap resampling were used for metrics with sufficient variability, and Wilson score intervals were applied when bootstrap resampling failed due to lack of variability. Statistical comparisons were performed using McNemar’s test (sensitivity/specificity) and Fisher’s exact test (PPV/NPV).

NA, not applicable; NAT, neoadjuvant therapy; NPV, negative predictive value; PPV, positive predictive value; TNBC, triple negative breast cancer.

STROBE Statement—Checklist of items that should be included in reports of ***cohort studies***

|  | Item No | Recommendation | Page No |
| --- | --- | --- | --- |
| **Title and abstract** | 1 | (*a*) Indicate the study’s design with a commonly used term in the title or the abstract | 1-2 |
|  |  | (*b*) Provide in the abstract an informative and balanced summary of what was done and what was found | 2 |
| Introduction | | | |
| Background/rationale | 2 | Explain the scientific background and rationale for the investigation being reported | 3 |
| Objectives | 3 | State specific objectives, including any prespecified hypotheses | 4 |
| Methods | | | |
| Study design | 4 | Present key elements of study design early in the paper | 9 |
| Setting | 5 | Describe the setting, locations, and relevant dates, including periods of recruitment, exposure, follow-up, and data collection | 9 |
| Participants | 6 | (*a*) Give the eligibility criteria, and the sources and methods of selection of participants. Describe methods of follow-up | 9-10 |
|  |  | (*b*) For matched studies, give matching criteria and number of exposed and unexposed | 9-10 |
| Variables | 7 | Clearly define all outcomes, exposures, predictors, potential confounders, and effect modifiers. Give diagnostic criteria, if applicable | 10-11 |
| Data sources/ measurement | 8* | For each variable of interest, give sources of data and details of methods of assessment (measurement). Describe comparability of assessment methods if there is more than one group | 10-11 |
| Bias | 9 | Describe any efforts to address potential sources of bias | 11 |
| Study size | 10 | Explain how the study size was arrived at | 9 |
| Quantitative variables | 11 | Explain how quantitative variables were handled in the analyses. If applicable, describe which groupings were chosen and why | 12 |
| Statistical methods | 12 | (*a*) Describe all statistical methods, including those used to control for confounding | 12 |
|  |  | (*b*) Describe any methods used to examine subgroups and interactions | 12 |
|  |  | (*c*) Explain how missing data were addressed | 9-10 |
|  |  | (*d*) If applicable, explain how loss to follow-up was addressed | 9-10 |
|  |  | (*e*) Describe any sensitivity analyses | 12 |
| Results | | |  |
| Participants | 13* | (a) Report numbers of individuals at each stage of study—eg numbers potentially eligible, examined for eligibility, confirmed eligible, included in the study, completing follow-up, and analysed | 4 |
|  |  | (b) Give reasons for non-participation at each stage | 4 |
|  |  | (c) Consider use of a flow diagram | 4 |
| Descriptive data | 14* | (a) Give characteristics of study participants (eg demographic, clinical, social) and information on exposures and potential confounders | 4 |
|  |  | (b) Indicate number of participants with missing data for each variable of interest | 4 |
|  |  | (c) Summarise follow-up time (eg, average and total amount) | 4 |
| Outcome data | 15* | Report numbers of outcome events or summary measures over time | 4 |

| Main results | 16 | (*a*) Give unadjusted estimates and, if applicable, confounder-adjusted estimates and their precision (eg, 95% confidence interval). Make clear which confounders were adjusted for and why they were included | 4-5 |
| --- | --- | --- | --- |
|  |  | (*b*) Report category boundaries when continuous variables were categorized | 4-5 |
|  |  | (*c*) If relevant, consider translating estimates of relative risk into absolute risk for a meaningful time period | Not applicable |
| Other analyses | 17 | Report other analyses done—eg analyses of subgroups and interactions, and sensitivity analyses | 6-7 |
| Discussion | | | |
| Key results | 18 | Summarise key results with reference to study objectives | 7 |
| Limitations | 19 | Discuss limitations of the study, taking into account sources of potential bias or imprecision. Discuss both direction and magnitude of any potential bias | 9 |
| Interpretation | 20 | Give a cautious overall interpretation of results considering objectives, limitations, multiplicity of analyses, results from similar studies, and other relevant evidence | 9 |
| Generalisability | 21 | Discuss the generalisability (external validity) of the study results | 9 |
| Other information | | | |
| Funding | 22 | Give the source of funding and the role of the funders for the present study and, if applicable, for the original study on which the present article is based | 13 |

*Give information separately for exposed and unexposed groups.

**Note:** An Explanation and Elaboration article discusses each checklist item and gives methodological background and published examples of transparent reporting. The STROBE checklist is best used in conjunction with this article (freely available on the Web sites of PLoS Medicine at http://www.plosmedicine.org/, Annals of Internal Medicine at http://www.annals.org/, and Epidemiology at http://www.epidem.com/). Information on the STROBE Initiative is available at http://www.strobe-statement.org.
